# Editorial: Public research and private knowledge—Science in times of diverse research funding

**DOI:** 10.3389/frma.2022.1106343

**Published:** 2022-12-15

**Authors:** Jon Leefmann, Julia Böttcher, Michael Jungert, Christoph Merdes, Sebastian Schuol

**Affiliations:** Center for Applied Philosophy of Science and Key Qualifications (ZiWiS), Friedrich-Alexander-Universität Erlangen-Nürnberg, Erlangen, Germany

**Keywords:** research funding, scientific integrity, values in science, social epistemology of science, research ethics, biased research

The production and distribution of knowledge is a key process in scientific and scholarly inquiry. However, this process is not and has never been limited to universities and public research institutes alone, but extends to agents as diverse as the Research & Development departments of companies, citizen scientists, and private non-profit research institutes. In recent years, these agents have shown an increased interest in basic science, in particular in fields of rising social significance such as AI or biomedical technologies. These interests in turn direct attention to the sources of funding and the interactions and collaborations between academic systems and the private sector. But, what difference does it make who funds research? Who are the relevant providers of funding? Does the influence of private funding change the selection of research topics in epistemically and ethically (un-)desirable ways? Does it lead to a privatization of knowledge, and if so, what are the consequences?

These questions unite the eight multidisciplinary contributions to this Research Topic. Comprised of six research papers and two critical perspectives, the issue offers a complex and multifaceted picture of the current debate on research funding at the intersection of research policy, philosophy, sociology, and science and literature studies. It also serves as a showcase for contributions that were presented and discussed at the international conference “*Public research and private knowledge—Science in times of diverse research funding*” organized by the Center for Applied Philosophy of Science (ZiWiS) at FAU Erlangen-Nürnberg from July 21st to July 23rd, 2021.

The papers in this Research Topic approach the subject from various theoretical backgrounds and by using examples from research areas as diverse as pharmacology, genetics, or literature—to name just a few—in order to reflect on the influence of funding sources on scientific practices. They can be broadly divided into three categories:

## Research funding and the integrity of scientific research

A first group of papers discusses the influence of diverse funding on the integrity of science. These empirically informed analyses of specific research practices argue that funding is often distributed in ways that are epistemically detrimental and ethically problematic. In one or another way they seek to reveal social mechanisms that explain and justify their claims. To this end they offer analyses of the Open Science movement (Fernández Pinto), the debate on policy regulations for genome editing (Christian), and the pharmaceutical industry's strategy of managing the processes of research and publication (Sismondo). All of them find that funding from the private sector plays a critical role in the establishment and maintenance of epistemically problematic practices.

## Biased assessment of private research funding

A second group of papers takes a critical stance toward these claims. Reviewing the literatures on private research funding in the philosophy of science and in research policy, one paper (Holman) finds that studies in research policy tend to evaluate relations between industry and academia primarily as beneficial, whereas the philosophy of science literature depicts such relations as generally corrosive for scientific inquiry. For a better assessment of the overall effect of industry funding on various research fields, it points to the origins of these contradicting perspectives and suggests venues for reaching a fruitful interaction of these distinct literatures. Similarly, another contribution (Sikimić) calls for a more nuanced take on the prospects and perils of industry-academic relations. Taking the example of different strategies to develop vaccines against COVID-19, it argues that funding schemes often do not fit the neat distinction between publicly and privately funded research. Publicly funded research can pose similar threats to the epistemic integrity of science as privately funded research and it may tend to promote elitism in science and the exclusion of research from institutions outside of Europe, Japan and North America. As the example of COVID-19 vaccine research shows, a perspective that is critical only about privately funded research is unduly simplistic.

## Bias and values in science

The third group of papers deals with the theoretical framework of debating the epistemic and ethical effects of research funding. Since the beginning of the 20th century, scientific enquiry has been described as a process of empirically testing hypotheses that is free from non-epistemic values, i.e., prudential, moral or political judgements. This view, however, has been under constant attack at least since Thomas Kuhn's seminal work (Kuhn, 1977/2000). More recently, drawing on debates in philosophy of science from the early 1950s, Heather Douglas and others have made the case that various stages of the research process require determining the distribution of inductive risks (Douglas, 2000). Deciding which risks are worth taking, however, requires evaluating the consequences of one's decision. If this is correct, the distinction between biased and unbiased research cannot simply be grounded in the distinction between value-laden and value-free scientific practices. Two papers in this section address this issue through discussing Torsten Wilholt's methodological conventionalism as a possible solution to this problem (Wilholt, 2009, 2013). While the first (Ohnesorge) launches a critique of this view based on theoretical and practical considerations, the second (Leefmann) defends it against a competing version of empiricism arguing for its superior capacity to explain how financial power can create biases by distorting otherwise helpful social practices.

Contrasting this debate, a further contribution (Hempel) suggests a different methodological approach. This paper combines literary studies (science in fiction) with a sociological perspective and discusses two contemporary science novels. By analyzing how the concepts of autonomy and responsibility of science become manifest in two novels which both deal with research misconduct in biomedical research, it explores cultural understandings of these concepts and studies how the depiction of science in popular culture can offer conceptual insights into social actors, actor constellations, and interactions within and beyond the institution of science. Thus, it addresses the issue of research funding by a new approach that provides a valuable resource for further sociological and philosophical analysis.

As topic editors we believe that this Research Topic will provide the reader not only with exemplary analyses of the epistemic and ethical dimensions of research funding but will also highlight new directions for promising research and encourage interaction between different methodological and disciplinary approaches to address the topic.

Finally, we would like to sincerely thank all the authors, reviewers and external editors who contributed to the creation and compilation of these research papers. We also thank Frontiers in Research Metrics and Analytics for technical support and for publishing this collection as part of their Research Topic series.

## Author contributions

All authors listed have made an intellectual contribution to the work and approved it for publication. The corresponding author's (JL) leadership in writing the editorial is acknowledged.
